# Solifenacin/Mirabegron Induces an Acute Compliance Increase in the Filling Phase of the Capacity-Reduced Urinary Bladder: A Pressure-Volume Analysis in Rats

**DOI:** 10.3389/fphar.2021.657959

**Published:** 2021-05-26

**Authors:** Hsien-Yu Peng, Cheng-Yuan Lai, Ming-Chun Hsieh, Tzer-Bin Lin

**Affiliations:** ^1^ Department of Medicine, Mackay Medical College, New Taipei City, Taiwan; ^2^ Department of Physiology, School of Medicine, College of Medicine, Taipei Medical University, Taipei City, Taiwan; ^3^ Cell Physiology and Molecular Image Research Center, Wan Fang Hospital, Taipei Medical University, Taipei City, Taiwan; ^4^ Department of Biotechnology, College of Medical and Health Science, Asia University, Taichung, Taiwan

**Keywords:** mirabegron, solifenacin, compliance, urinary bladdder, pressure-volume curve

## Abstract

**Aims:** Pressure in the bladder, which is a high compliance organ, is only slightly elevated to a considerable filling volume during storage. Although cystometry off-line offers mean compliance, no protocol is available for real-time assays of the dynamics of bladder compliance, and the potential impact of solifenacin and mirabegron on dynamic bladder compliance has not been established.

**Methods:** Along with constantly infused cystometry, a pressure-volume analysis (PVA) was performed by plotting intra-vesical volume against pressure in Sprague-Dawley rats. The instant compliance was assayed as the slope of the trajectory, and the mean compliance (Cm) was determined by the slope of the line produced by regression of the data points at the end of the first, second, and third quarters of the filling phase.

**Results:** Under a steady-state, the PVA trajectory moved clockwise which shaped coincident enclosed loops with stable compliance. Though administering to naïve animals solifenacin, but not mirabegron (both 1 × 10^−5^−1 × 10^−1^ mg/kg, i.a.) decreased the peak pressure, both of these reagents exhibited acute increments in the trajectory slope and Cm of the filling phase in a dose-dependent manner (ED_50_ = 1.4 × 10^−4^ and 2.2 × 10^−5^ mg/kg, respectively). Resembling urine frequency/urgency in OAB patients, the voiding frequency of a capacity-reduced bladder was increased in association with decreased compliance which was ameliorated by both acute solifenacin and mirabegron injections (both 1 × 10−1 mg/kg).

**Conclusion:** In addition to their well-known anti-inotropic/relaxative effects, solifenacin, and mirabegron induce an acute increase in bladder compliance to ameliorate OAB-like syndromes. Together with time-domain cystometry, PVA offers a platform for investigating the physiology/pathophysiology/pharmacology of bladder compliance which is crucial for urine storage.

## Introduction

The urinary bladder collects and stores urine before its disposal by urination (De Groat and Yoshimura, 2001); and the pressure of the bladder, which is a highly compliant organ, is only slightly elevated in response to a considerable filling volume during storage (Sugaya et al., 2000). Impaired bladder compliance is deleterious because abnormally high intra-vesical pressure (IVP) not only obstructs urine flow to cause vesico-urethral reflux (Wu and Franco, 2017) but also overactivates the micturition neuraxis (Mills et al., 2000) to underlie the development of an overactive bladder (OAB) manifested by urinary urgency and/or frequency (Ali et al., 2019).

Although no protocol is currently available for the real-time assessment of bladder compliance to the best of our knowledge, urodynamic studies off-line analyze the Cm of the filling phase by dividing bladder capacity by the pressure difference between the threshold and baseline pressures (Cameron et al., 2009). Although this method offers a mean value of compliance over the entire filling phase, to explore dynamic compliance changes during the filling phase seems difficult; and thereby to specifically investigate compliance stages of interest, e.g., at a critical pressure triggering voiding or the minimal volume immediately after voiding, is still a challenge. Notably, a very recent study demonstrated that pressure-volume analysis (PVA) assays bladder functions in real-time (Peng et al., 2020), which thus offers the chance to analyze the relationship between bladder pressure and volume on-line. In this study, we aimed to test whether PVA is able to assess compliance dynamics of the bladder on-line, and hence to provide benefits to basic/clinical studies investigating bladder disorders by focusing on storage impairments.

Mirabegron is recognized for its ability to relax bladder smooth muscle by selectively binding to β3-adrenergic receptors (Imamura et al., 2017). On the other hand, solifenacin diminishes detrusor contractility by antagonizing M3 receptors, the primary receptor mediating detrusor contraction (Furuta et al., 2016). In clinical practice, solifenacin and mirabegron are prescribed to OAB patients who present problems of urine storage manifesting as urinary urgency and/or frequency (Gratzke et al., 2019). Although both of these drugs display satisfactory symptom relief by reducing urgency episodes and urinary frequency (Simpson and Wagstaff, 2005; Chapple et al., 2020); and studies measuring Cm using cystometry have shown that mirabegron (Krhut et al., 2018) and solifenacin (Franco et al., 2020) increase Cm of the bladder in patients, the detailed therapeutic benefits on the compliance dynamics have not been established. We hence tested the potential impact of mirabegron and solifenacin on PVA-derived compliance dynamics.

## Materials and Methods

### Animal Preparations

The animal procuration/husbandry/experiments conformed to the “European Convention for the Protection of Vertebrate Animals used for Experimental and other Scientific Purposes”. Experiments were reviewed and approved by the Institutional Review Board of Taipei Medical University, Taipei, Taiwan (LAC-2019–0509 in institutional animal care and use committee, Taipei Medical University). Forty-five adult female Sprague-Dawley rats (200–300 g; BioLASCO, Taipei, Taiwan) were assigned randomly to experiment groups with sample sizes set before data acquisition.

### Surgery/Cystometry Procedures

After being anesthetized with subcutaneous urethane (1.2 g/kg), two catheters were placed into the left jugular vein and femoral artery for injecting supplemental anesthetics and drugs, respectively. Following a midline laparotomy, ureters were bilaterally transected and drained freely. A wide-bore cannula, which was tied into the bladder lumen through an incision on the apex of the bladder dome, was connected *via* a three-way stopcock to a syringe pump and a pressure transducer (P231D, Gould-Statham, Quincy, IL) for saline infusing and IVP recording, respectively. After emptying, saline with a constant rate (0.04–0.08 ml/min) was continuously infused into the bladder to provoke voiding contractions. The accumulated volumes of infused and voided fluid were continuously recorded by measuring the weight of fluid using strain gauges (FT03C GRASS, West Warwick, RI). In some experiments, a suture thread placed at the level about 1/3 of the bladder length below the bladder dome was secured to reduce bladder contents (Supplementary Figure S1A). Urodynamic parameters including peak pressure (the maximal IVP), post-voiding pressure (the minimal IVP after voiding), voiding frequency (the count per min), and voided volume (the expelled fluid volume) of voiding contractions were analyzed when tracings displayed at least three uniformed contractions.

### Pressure-Volume Analysis

Protocols for measuring intra-vesical volume (IVV) and IVP were adapted from a previous publication (Peng et al., 2020); nevertheless, in contrast to that study, which aimed to measure voiding work by plotting IVP against IVV, we reversed the abscissa with coordinates, i.e., plotted IVV against IVP based on the definition of compliance (Nyirady et al., 2002). Dynamic bladder compliance was assayed as the slope of the trajectory during the filling phase; and the Cm was calculated using the slope of the line determined by regression of the data points at the end of the first, second, and third quarters of the filling phase (Supplementary Figure S1B).

### Drugs Administration

Solifenacin (solifenacin succinate; dissolved in ddH2O) and mirabegron (dissolved in 8% alcohol) were injected into animals (1 × 10^−5^−1 × 10^−1^ mg/kg, bolus; both Sigma-Aldrich, Shanghai, China) *via* an implanted intra-arterial catheter. Solvents of these reagents were used as vehicle controls.

### Statistical Analysis

All data, which were averaged from 2–3 continuous voiding cycles, were analyzed using SigmaPlot (Systat Software, Chicago, IL) and were expressed as mean ± SEM. After checking the normality and variance using Shapiro-Wilk and equal variance tests, respectively, one-way ANOVA was used to assess the difference between groups; and a post-hoc Student-Newman-Keuls test was used to compare the means of groups when an adequate F ratio was achieved. Statistical significance was assigned at *p* < 0.05.

## Results

### Pressure-Volume Analysis-Derived Bladder Compliance

**FIGURE 1:**
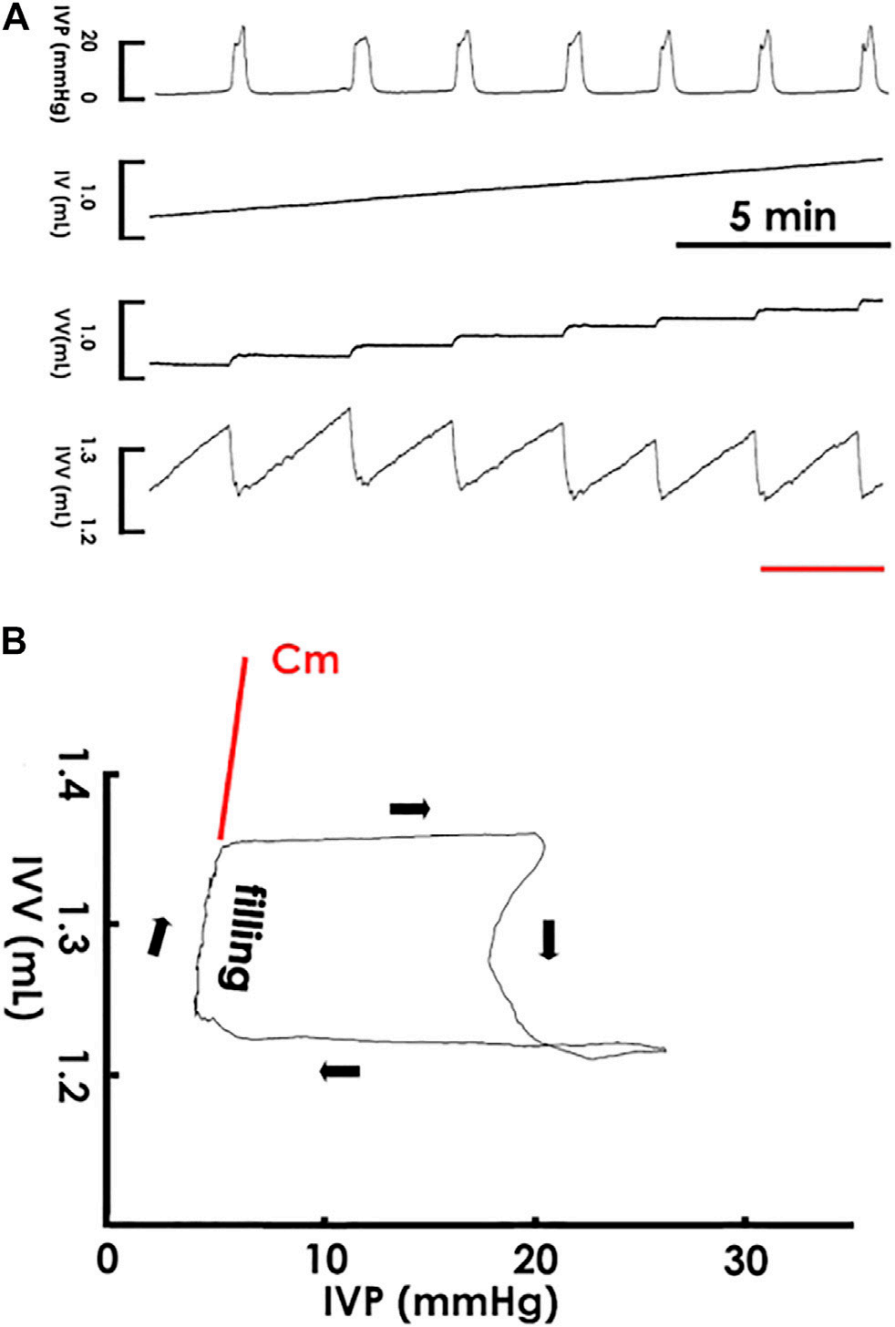
Cystometry and pressure-volume analysis. **(A)** Cystometry showing that saline infusion (0.04 ml/min) into the bladder provokes rhythmic voiding contractions with a constant frequency as well as uniformed peaks of IVP and fluctuations of intra-vesical volume (IVV). The red bar at the bottom marks the cycle displayed in the pressure-volume analysis. **(B)** A pressure-volume analysis established by plotting IVV against IVP. The data trajectory moves clockwise and shapes an enclosed loop. The left bolder of the loop represents the filling phase, and the instantaneous slope of the trajectory reflected the dynamic compliance at specific points of the filling phase. The slope of the regression line of the filling trajectory denotes the mean compliance (Cm) of a loop. IV, infused volume; VV, voided volume.

Cystometry showed that a constant saline infusion into the pre-emptied bladder provoked rhythmic voiding contractions (Figure 1A); and in the pressure-volume analysis (PVA), which plotted intra-vesical volume against pressure (IVV and IVP, respectively), the data trajectory moved clockwise, which shaped enclosed loops representing voiding cycles (Figure 1B). The left border of the loops, which displayed a progressively increased IVV with a slightly elevated IVP, characterized the filling phase, and the instantaneous slope of the trajectory, which denoted the change in IVV divided by a corresponding change of IVP in specific points, reflected the compliance dynamics of the filling phase.

### Baseline Compliance Remains Stable

**FIGURE 2:**
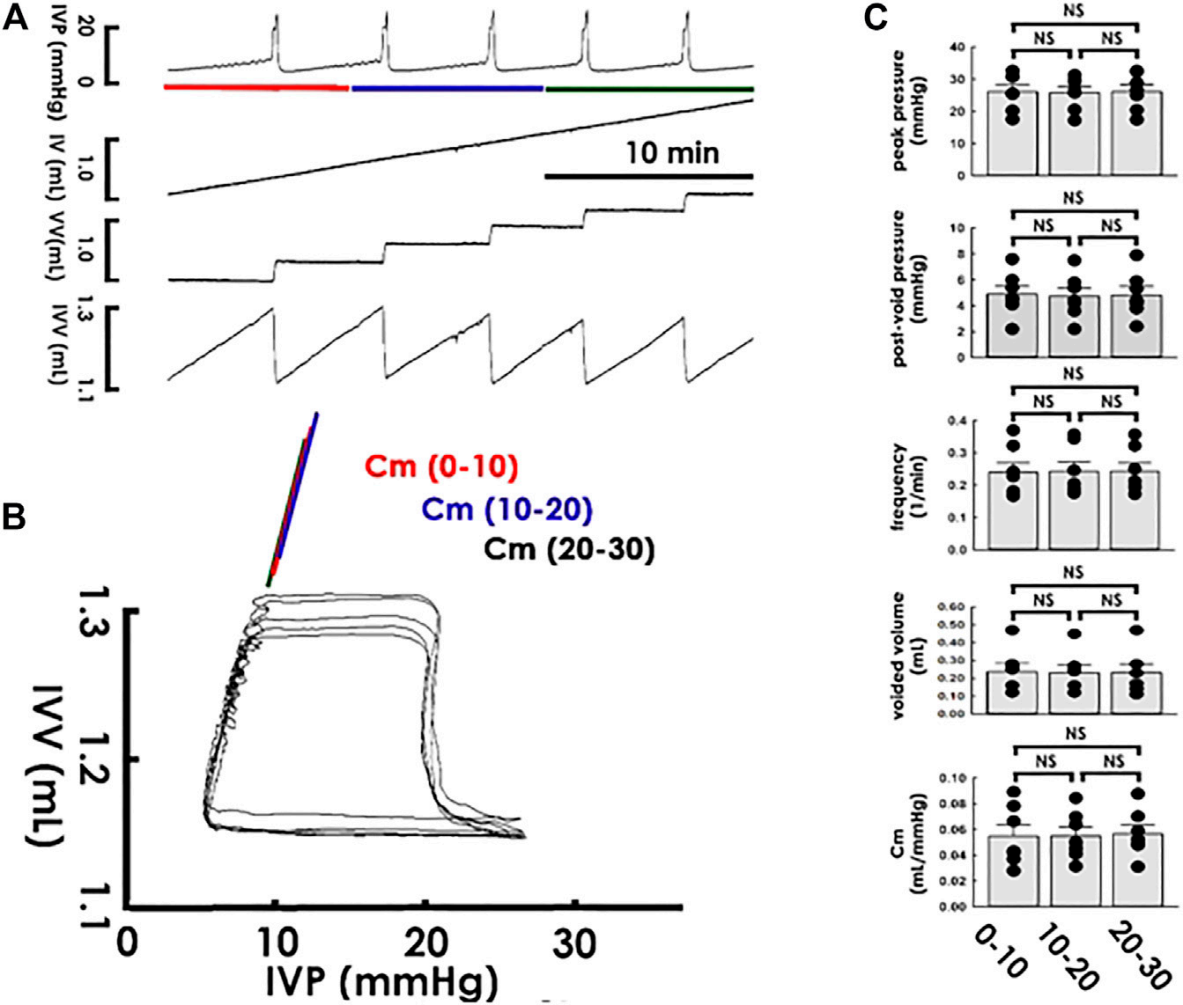
Cystometry and pressure-volume analysis of rhythmic voiding contractions. **(A)** Cystometry showing regular voiding contractions with a constant frequency at a period of 30 min. Red, blue, and green bars at the bottom of IVP mark the cycles displayed in the pressure-volume analysis. **(B)** Pressure-volume analysis showing overlapped loops. The mean compliance (Cm) of loops at the first (0–10; red), second (10–20; blue), and last (20–30; green) 10 min are well-coincident. **(C)** No statistical difference is evidenced in the peak pressure, post-void pressure, voiding frequency, voided volume, and Cm between contractions of the first, second, and last 10 min of the recording period. IVP, intra-vesical pressure; IV, infused volume; VV, voided volume; IVV, intra-vesical volume.

After establishing PVA to assay compliance dynamics, we tested the reliability of PVA-derived compliance by examining if it remains stable in a steady state. In accompaniment with the cystometry, it displayed that a constant saline infusion provoked uniformed voiding contractions with a constant frequency within 30 min (Figure 2A), PVA showed coincident loops with well-overlapping trajectories in the filling phase (Figure 2B). To further confirm the consistency of PVA-derived compliance, the Cm of the loops was calculated using the slope of the line determined by regression of the data points at the end of the first, second, and third quarters of the filling phase. Analogously to the summarized data, this demonstrated that cystometric parameters, including the peak pressure, post-voiding pressure, voiding frequency, and voided volume, remained statistically unchanged (Figure 2C; all *p* > 0.05 vs. each other, *n* = 7), the Cm of the PVA loops displayed no significant difference between the first (0–10), second (10–20), and last (20–30) 10 min (all *p* > 0.05 vs. each other, *n* = 7).

### Solifenacin Induces an Acute Compliance Increase

**FIGURE 3:**
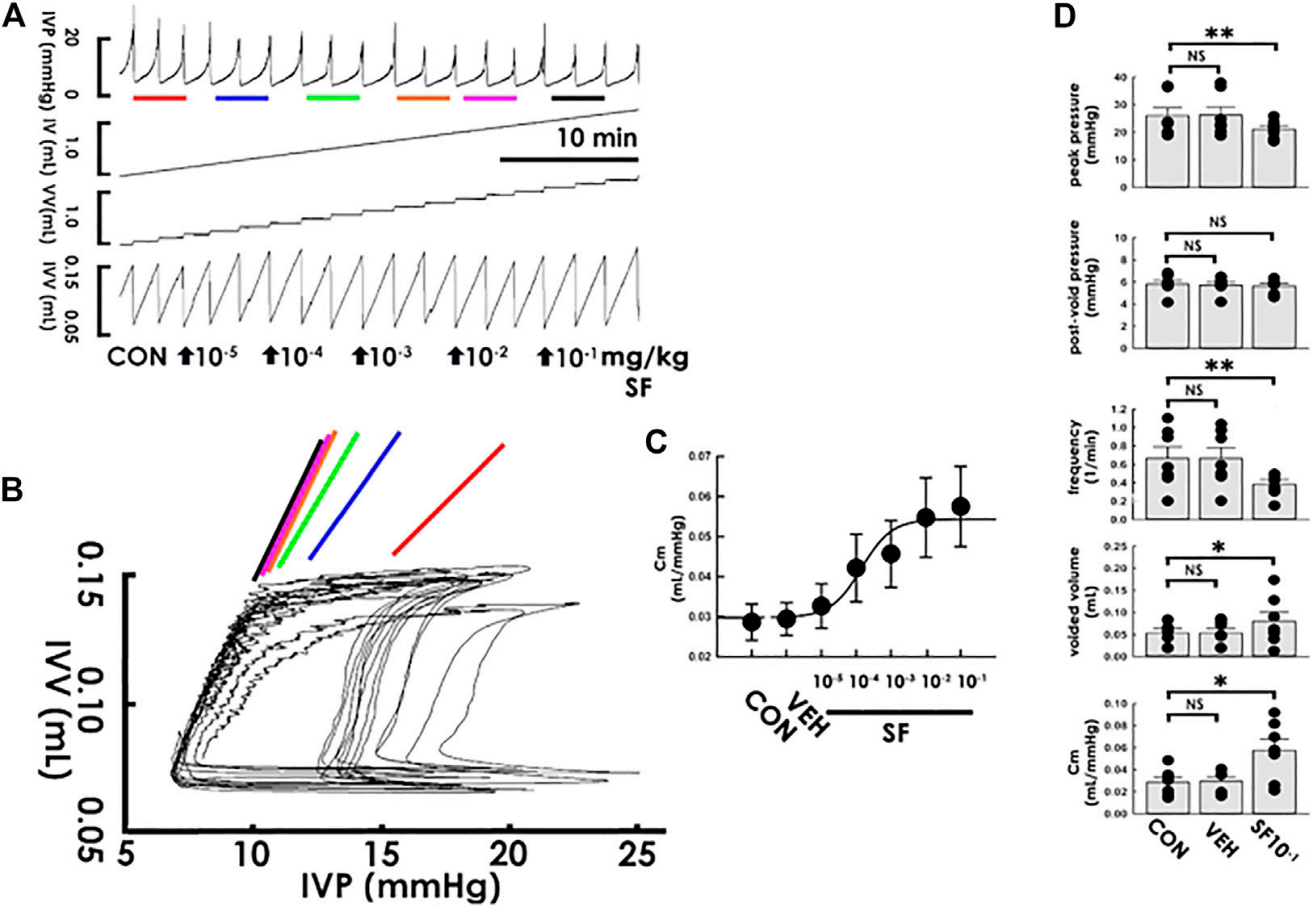
Solifenacin increases bladder compliance. **(A)** Cystometry before (control; CON) and following intra-arterial solifenacin (SF) injections with increasing concentrations (10^−5^−10^−1^ mg/kg, bolus). Red, blue, green, orange, pink, and black bars at the bottom of IVP mark the cycles displayed in the pressure-volume analysis. **(B)** Pressure-volume loops showing increasing concentrations of solifenacin progressively increases the slope of the regression line of the filling phase. **(C)** Dose-response analysis showing solifenacin step-wisely increases the mean compliance (Cm) with an ED50 at about 1.4 × 10^−4^ mg/kg. **(D)** While vehicle solution (VEH) displays no statistical difference in parameters, injection of solifenacin (1 × 10^−1^ mg/kg; SF1X10^−1^) significantly decreases the peak pressure and voiding frequency but increases voided volume and Cm (*p < 0.05, **p < 0.01, vs. CON; all *n* = 7). IVP, intra-vesical pressure; IV, infused volume; VV, voided volume; IVV, intra-vesical volume.

Observing that the PVA-derived compliance remained stable under a steady state, we tested the acute impacts of solifenacin, an M3 antagonist proven to treat symptoms, on compliance dynamics by focally administering naïve animals solifenacin through a femoral artery catheter (1 × 10^−5^−1 × 10^−1^ mg/kg, bolus). In addition to reducing the peak pressure in the cystometry (Figure 3A), administering solifenacin at increased concentrations progressively elevated the slope of the filling trajectory in PVA (Figure 3B), an effect confirmed by the dose-response analysis showing that solifenacin stepwise increased Cm with an ED_50_ of approximately 1.4 × 10^−4^ mg/kg (Figure 3C). In contrast to the vehicle solution, which exhibited no effect (Figure 3D VEH; all *p* > 0.05 vs. control, *n* = 7), the summarized data demonstrated that solifenacin (1 × 10^−1^ mg/kg) significantly decreased peak pressure and voiding frequency but increased the voided volume and Cm (SF10^−1^; all DF = 2, F = 7.06, 13.40, 8.53, and 10.45, respectively, all *p* < 0.01 vs. control, *n* = 7) without affecting post-void pressure (*p* > 0.05 vs. control, *n* = 7).

### Mirabegron Induces an Acute Compliance Increase

**FIGURE 4:**
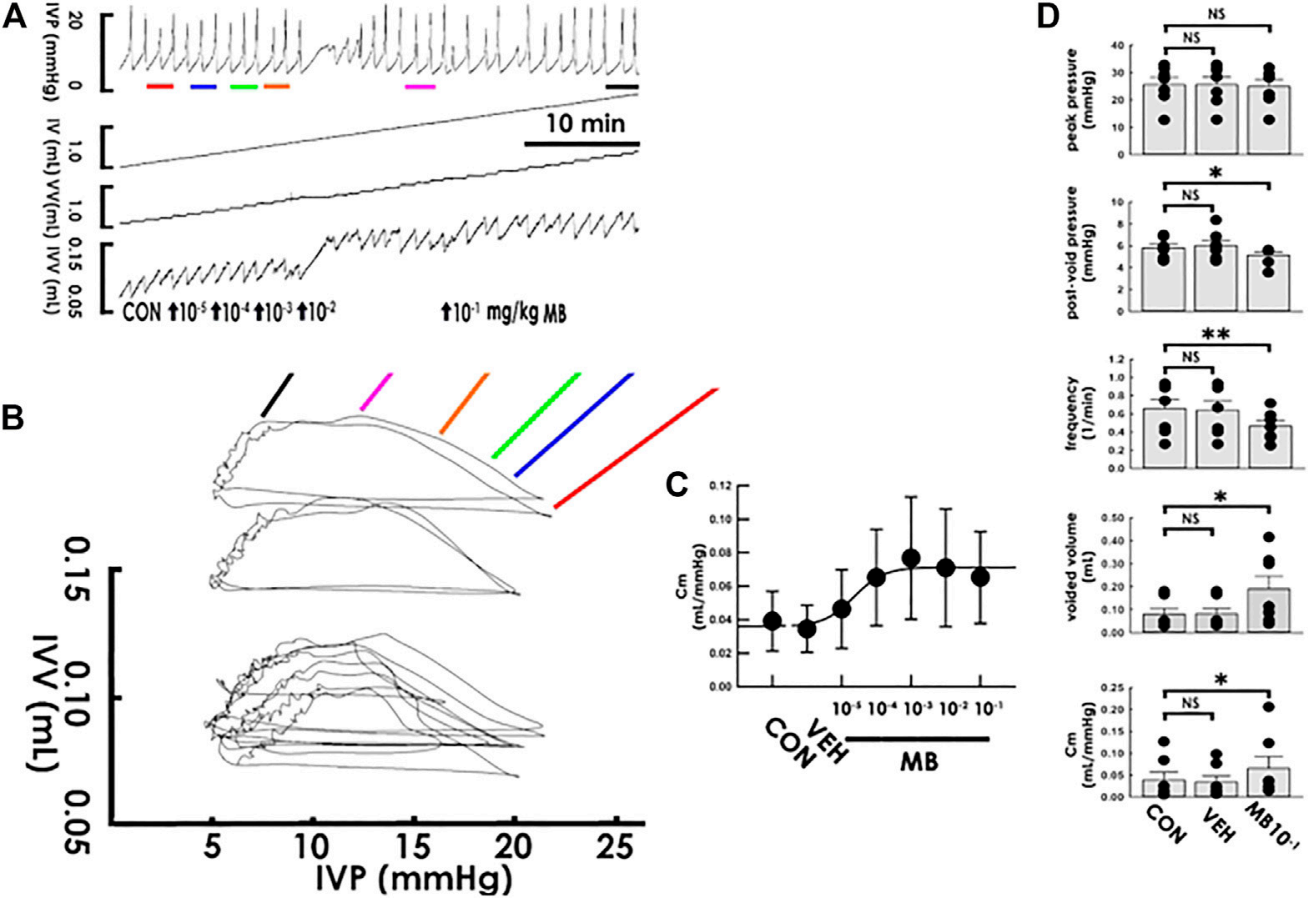
Mirabegron increases bladder compliance. **(A)** Cystometry before (control; CON) and following intra-arterial mirabegron (MB) injections with increasing concentrations (10^−5^−10^−1^ mg/kg, bolus). Red, blue, green, orange, pink, and black bars at the bottom of IVP mark the cycles displayed in the pressure-volume analysis. **(B)** Pressure-volume loops showing increasing concentrations of mirabegron progressively increases the slope of the regression line of the filling phase. **(C)** Dose-response analysis showing mirabegron step-wisely increases the mean compliance (Cm) with an ED_50_ at about 2.2 × 10^−5^ mg/kg. **(D)** While vehicle solution (VEH) displays no statistical difference in parameters, injection of mirabegron (1 × 10^−1^ mg/kg; MB1X10^−1^) significantly decreases the post-void pressure and voiding frequency but increases voided volume and Cm (*p < 0.05, **p < 0.01, vs. CON; all *n* = 7). IVP: intra-vesical pressure, IV: infused volume, VV: voided volume, IVV intra-vesical volume.

Next, the acute effects of mirabegron, a beta-3 agonist also widely prescribed for treating OAB, on compliance dynamics was like-wise investigated. Without markedly affecting the peak pressure in the cystometry (Figure 4A), focal mirabegron injections (1 × 10^−5^−1 × 10^−1^ mg/kg, i.a., bolus) with increased concentrations gradually increased the slope of the filling trajectory in the PVA (Figure 4B). Dose-response analysis showed that mirabegron stepwise increased the Cm with an ED_50_ of approximately 2.2 × 10^−5^ mg/kg (Figure 4C). Moreover, while the vehicle solution displayed no effect (Figure 4D VEH; all *p* > 0.05 vs. control, *n* = 7), the summarized data demonstrated that mirabegron (1 × 10^−1^ mg/kg) significantly decreased post-void pressure and voiding frequency but increased the voided volume and Cm (MB10^−1^; all DF = 2, F = 5.55, 9.28, 6.78, and 5.35; *p* < 0.05, <0.01, *p* < 0.05, and *p* < 0.05, respectively, vs. control, *n* = 7) without affecting peak pressure (*p* > 0.05 vs. control, *n* = 7).

### Acute Solifenacin Injection Ameliorates Restriction-Decreased Compliance

**FIGURE 5:**
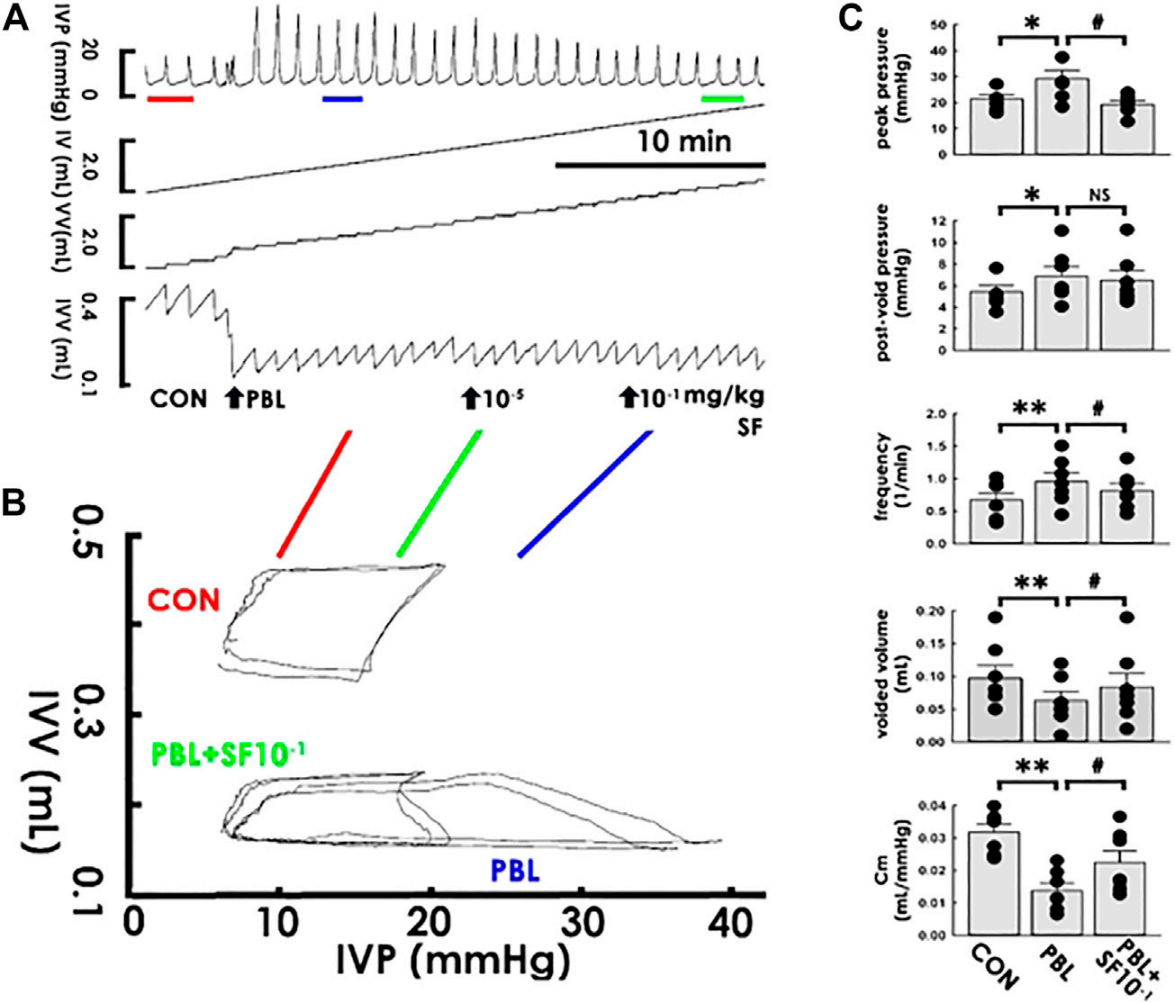
Solifenacin ameliorates bladder ligation-reduced compliance. **(A)** Cystometry before (control; CON) and in response to a partial bladder ligation (PBL) followed by intra-arterial injections of solifenacin (SF; 10^-5^ and 10^−1^ mg/kg, bolus). Red, blue, and green bars at the bottom of IVP mark the cycles displayed in the pressure-volume analysis. **(B)** Pressure-volume loops showing a partial bladder ligation reduces the slope of the regression line of the filling phase that is marked reversed by solifenacin with a concentration of 10^−1^ mg/kg (PBL + SF10^−1^). **(C)** A partial bladder ligation statistically increases the peak pressure, post-void pressure, voiding frequency but decreases voided volume and mean compliance (Cm; *p < 0.05, **p < 0.01, vs. CON; all *n* = 7) that are all ameliorated by solifenacin (1 × 10^−1^ mg/kg; SF1X10^−1^) excepting the post-void pressure (^#^ *p* < 0.05, vs. PBL; all *n* = 7). IVP, intra-vesical pressure; IV, infused volume; VV, voided volume, IVV intra-vesical volume.

Apprehending that solifenacin induces an acute compliance increment under a physiological state, we next investigated if solifenacin exhibits therapeutic benefits also to pathophysiological conditions by examining its acute impact on the compliance dynamics of a capacity-restricted bladder. Accompanied with a marked increased peak pressure but a decreased voided volume, acute partial bladder ligation (PBL) increased the voiding frequency in the cystometry (Figure 5A), which resembled urinary frequency/urgency in OAB patients. In PVA, PBL distinctly decreased the slope of the filling trajectory (Figure 5B). Though solifenacin at 1 × 10^−5^ mg/kg displayed no obvious effect, administering PBL animals with solifenacin of 1 × 10^−1^ mg/kg (both bolus, i.a.) not only ameliorated the PBL-increased peak pressure and voiding frequency as well as the PBL-decreased voided volume in the cystometry, but also alleviated the PBL-decreased slope of the filling trajectory in PVA. The summarized data demonstrated that PBL significantly increased peak pressure, post-void pressure, and voiding frequency but decreased the voided volume and Cm (Figure 5C; all DF = 2, F = 6.93, 6.18, 14.98, 11.68, and 11.04; *p* < 0.05, *p* < 0.05, *p* < 0.01, *p* < 0.01, and *p* < 0.01, respectively, vs. control, all *n* = 7). Focal solifenacin injection (1 × 10^-1^ mg/kg) significantly reversed the increased peak pressure and voiding frequency as well as decreased the voided volume and Cm (all *p* < 0.05 vs. PBL, *n* = 7) but exhibited no effect on the elevated post-void pressure (*p* > 0.05 vs. PBL, *n* = 7) induced by PBL.

### Acute Mirabegron Injection Ameliorates Restriction-Decreased Compliance

**FIGURE 6:**
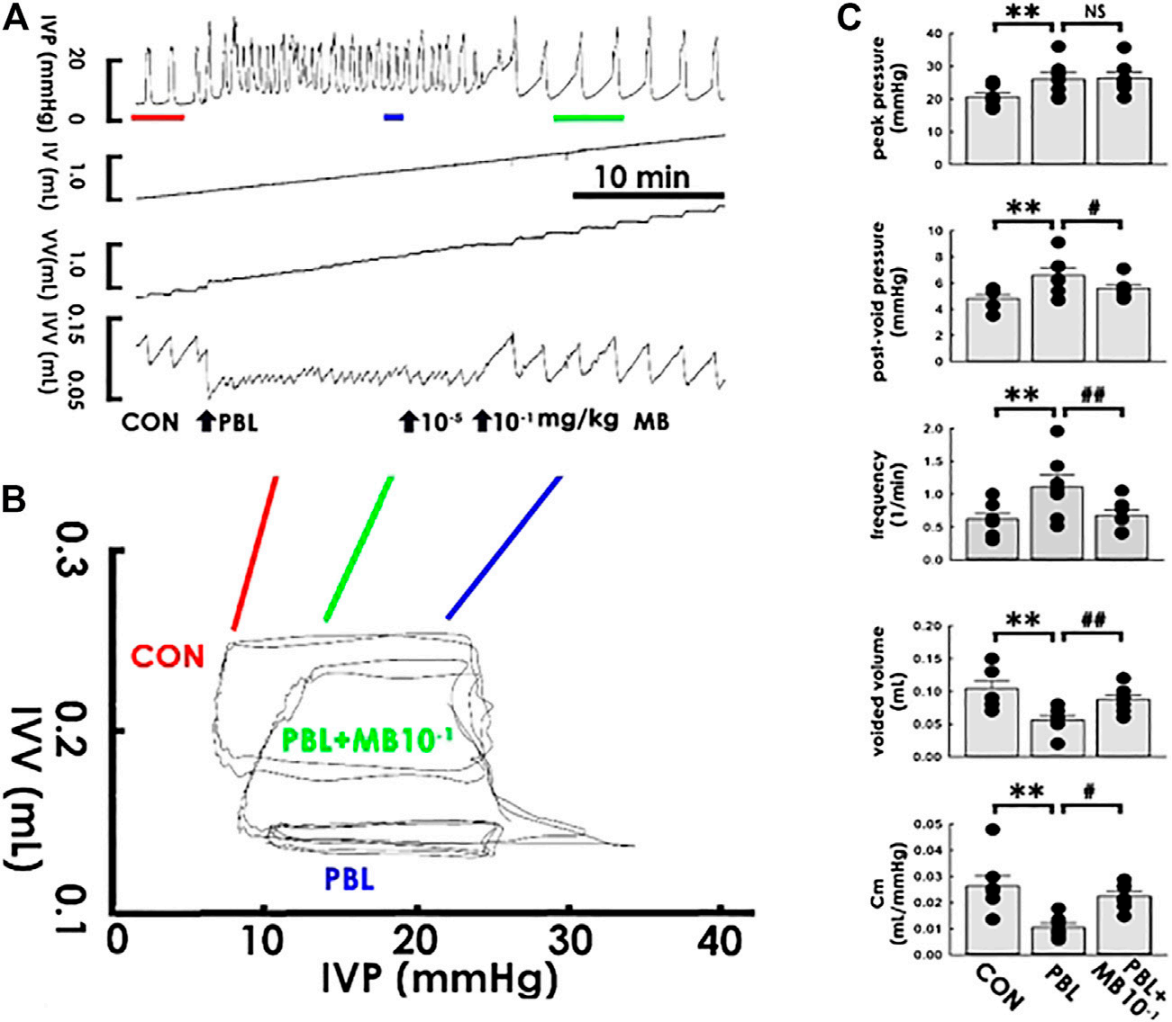
Mirabegron ameliorates bladder ligation-reduced compliance. **(A)** Cystometry before (control; CON) and in response to a partial bladder ligation (PBL) followed by intra-arterial injections of mirabegron (MB; 10^−5^ and 10^−1^ mg/kg). Red, blue, and green bars at the bottom of IVP mark the cycles displayed in the pressure-volume analysis. **(B)** Pressure-volume loops showing a partial bladder ligation reduces the slope of the regression line of the filling phase that is marked reversed by mirabegron with a concentration of 10^-1^ mg/kg (MB + SF10^−1^). **(C)** A partial bladder ligation statistically increases the peak pressure, post-void pressure, voiding frequency but decreases voided volume and mean compliance (Cm; **p < 0.01, vs. CON; all *n* = 7) that are all ameliorated by mirabegron (10^−1^ mg/kg) excepting the peak pressure (^#^ *p* < 0.05, ^##^ *p* < 0.01 vs. PBL; all *n* = 7).

The potential therapeutic benefits of acute mirabegron injection to OAB-like pathophysiological conditions were next correspondingly tested. While mirabegron at 1 × 10^−5^ mg/kg failed to exhibit a noticeable effect, mirabegron at 1 × 10^−1^ mg/kg (both bolus, i.a.) reversed the PBL-increased voiding frequency and—decreased voided volume in the cystometry (Figure 6A) as well as alleviated the PBL-decreased slope of the filling trajectory in the PVA (Figure 6B). The summarized data demonstrated that focal mirabegron injection (1 × 10^−1^ mg/kg) significantly reversed the increased post-void pressure and voiding frequency as well as decreased the voided volume and Cm (Figure 6C MB10^−1^; all DF = 2, F = 10.31, 7.87, 14.27, and 7.95; *p* < 0.05, *p* < 0.01, *p* < 0.01, and *p* < 0.05, respectively, vs. PBL, all *n* = 7) but displayed no effect on the elevated peak pressure (*p* > 0.05 vs. PBL, *n* = 7) caused by PBL.

## Discussion

### Pressure-Volume Analysis-Derived Compliance Benefits Scientists/Clinicians

During continence, the urinary bladder functions as a compliant reservoir that stores urine without producing marked pressure increments (Panicker and Fowler, 2010). Impaired bladder compliance fails to adequately store urine, which could lead to urine frequency, urgency, and incontinence (German et al., 1994). In contrast to *in vitro* studies evaluating bladder compliance by analyzing the length-tension relationship of bladder strips (Dean et al., 1997), *in vivo* investigations off-line assay compliance using cystometry by dividing the IVV changes by IVP changes during the filling phase (Cameron et al., 2009). Nevertheless, cystometry-derived compliance offers only a value of Cm based on the hypothesis that the compliance remains constant during the entire filling phase; therefore, specifically inspecting compliance of stages of interest, such as the early stage of the filling or critical stage before triggering a voiding contraction, seems not easy. Moreover, off-line analysis is time-consuming because it requires waiting for processing after laboratory investigations that seems inconvenient, particularly in clinical scenarios. Besides, technically, the IVP increase in the filling phase in time-domain cystometry is trivial and requires careful data acquisition/analysis by urodynamics experts.

In this study, we used real-time PVA to investigate bladder compliance. Instead of an averaged value, PVA monitored bladder compliance on-line, thereby displaying live compliance dynamics during filling. This protocol not only offers a chance to specifically inspect the compliance physiology/pathophysiology periods of interest but is also able to identify if there are changes in compliance during filling. Although this is not the usual case, we observed that compliance markedly increased in the early period of filling compared with that in the late period in a long-term distended bladder (unpublished data). Also, with advancements in computer technology, simultaneously displaying cystometry and PVA is no longer a challenge. On-line displayed compliance could provide immediate monitoring of compliance calculated by the computer with minimal calculation performed by the clinicians/scientists. Moreover, as the data in this study show, the trajectory of PVA visibly illustrates compliance with a satisfactory resolution that can easily be acquired by clinicians/scientists. Collectively, in accompanied with the ongoing cystometry, PVA provides a real-time and specific assessment of compliance dynamics with a good resolution that can be easily acquired by physicians/investigators; thereby, PVA could be used in clinical urodynamic investigations for diagnosis of impaired storage functions.

### Solifenacin/Mirabegron Increases Compliance

Solifenacin and mirabegron are widely prescribed to treat syndromes of OAB patients because solifenacin inhibits detrusor contraction (Cardozo et al., 2004) and mirabegron relaxes detrusors (Yeowell et al., 2018). Cystometry studies have revealed that both mirabegron (Krhut et al., 2018) and solifenacin (Madhuvrata et al., 2012; Franco et al., 2020) increase the Cm of the bladder in patients. Using PVA, our data in this study demonstrated that administering naïve animals with solifenacin and mirabegron both induced acute increases in the dynamic and mean compliances of the filling phase. Not restricted to a physiological condition, in a capacity-restricted bladder, solifenacin or mirabegron ameliorated PBL-decreased dynamic/Cm, indicating that these medications also exhibited effects under pathological conditions. Notably, accompanied with decreasing compliance in PVA, cystometry demonstrated that PBL increased voiding frequency, which resembles urine frequency/urgency in OAB patients, and acute administration of solifenacin or mirabegron both effectively alleviated the PBL-increased voiding frequency. Collectively, these results suggest that along with their well-recognized anti-inotropic and relaxative effects on the detrusor, at least in the case of capacity-restricted bladders, solifenacin, and mirabegron ameliorate OAB-associated urine frequency/urgency also by inducing an acute compliance increment, i.e., the bladder exhibited a lower pressure increment to urine accumulation that allows the bladder to store more fluid before triggering voiding.

Though our data demonstrated that solifenacin and mirabegron exhibited a similar effect on bladder compliance, the possibility that any reagents tested using this protocol could induce the same effect is not likely for the following reasons: 1) administering naïve animals with neither vehicle solutions of solifenacin (ddH2O) nor that of mirabegron (8% alcohol) displayed effect on dynamic/Cm, 2) dose-response analysis demonstrated that solifenacin and mirabegron exhibited different ED_50_ of Cm in naïve animals, 3) low-dose solifenacin/mirabegron failed to display discernable therapeutic effects in PBL animals; and notably, 4. in naïve and PBL animals, solifenacin diminished peak pressure without affecting post-void pressure, and conversely, mirabegron decreased post-void pressure without affecting peak pressure. These findings collectively suggested even though solifenacin and mirabegron pharmacologically exhibited different effects on the detrusor, namely impedes active detrusor contraction (Chilman-Blair and Bosch, 2004) and facilitates relaxation (Hatanaka et al., 2013; Sadananda et al., 2013), respectively, both of these drugs increased bladder compliance to relieve urine frequency/urgency. Our finding is consistent with clinical observations showing that even though mirabegron and solifenacin display different therapeutic and side effects, these reagents exhibit satisfactory symptom relief of urinary frequency and urgency (Gratzke et al., 2018).

Though it is a limitation that the results of this study did not clarify whether the anti-inotropic/relaxative effects on detrusors exhibit more pronounced therapeutic benefits than compliance increments, or quite the reverse, with the latter being much more important than the former, or whether both of these effects are basically equivalent because the elastance is a reciprocal of compliance, and a relaxation-reduced elastance is always associated with compliance increments ( German et al., 1994). Nevertheless, diminishing detrusor contractility does not seem to be a practical therapeutic strategy because it has the potential to impair the tension development essential for urine expulsion (Sugaya et al., 2000). Instead of focusing on detrusor contractility, our study specifically assayed the compliance, an explicit target for exploring storage functions, provided evidence supporting the therapeutic benefits of solifenacin/mirabegron in OAB-associated urine frequency/urgency.

In the current study, only the trajectory of the filling phase was analyzed; nevertheless, plotting a whole loop of voiding cycles would provide more information other than compliance. For example, solifenacin markedly shifted the right bolder of the loop to the left indicating a decreased peak voiding pressure possibly due to its anti-inotropic effect. On the other hand, with minimal effect on the right bolder, mirabegron shifted the loop upward, revealing an increased residual volume that might be caused by enhanced bladder relaxation. However, detailed correlations between PVA and cystometry need further studies to be established.

### Limitations in Pressure-Volume Analysis Investigations

Even though the etiology of urinary frequency/urgency in OAB patients seems to be multiple, studies have suggested lesions in the visco-elastic property (Cullingsworth et al., 2020) and contractility (Li et al., 2019) of the bladder as well as enhanced excitability in bladder sensory endings (Meng et al., 2012) and the nervous system in the lumbosacral spinal cord (Reynolds et al., 2016) and pontine micturition centers (Zorba et al., 2013) could underlie the development of symptoms. In addition to their well-established effects on the detrusor, the results of the present study revealed that the therapeutic effects of solifenacin and mirabegron on OAB-like symptoms could be attributed to an increased compliance, the visco-elastic property of the bladder itself. Nevertheless, because solifenacin and mirabegron are shown to exhibit their effects not restrictively on the detrusor but also on the urothelium, local perfusion, and afferent/afferent arms/centers of neural circuitry (Igawa et al., 2019) the potential role of these candidates in solifenacin/mirabegron-increased compliance needs further investigations.

To convert dosage from animal to human studies is important for safe and effective drug administration. Based on the normalization of dosage to body surface area, allometric scaling is used for dose conversion between animals and humans (Nair and Jacob, 2016). Because the mean body weight of animals in this study was within the range of this method, and the ED_50_s of solifenacin and mirabegron were 1.4 × 10^−4^ and 2.2 × 10^−5^ mg/kg; thereby the estimated human equivalent doses were approximately 2.2 × 10^−5^ and 3.5 × 10^−6^ mg/kg, respectively. Though these doses are markedly lower than the recommended doses of oral administration in clinical practices (Chilman-Blair and Bosch, 2004; Gratzke et al., 2018; Krhut et al., 2018; Franco et al., 2020), these equivalent doses could provide a reference for studies aiming to investigate storage of the bladder using focal drug injections.

## Conclusion

Accompanied with well-developed cystometry, we developed PVA-derived compliance as a promising protocol for investigating storage functions *in vivo*. Our results reveal that solifenacin and mirabegron also exhibit therapeutic benefits for OAB-associated symptoms by increasing bladder compliance. Our findings provide an additional therapeutic basis of solifenacin and mirabegron in treating OAB patients in clinical practice. On the other hand, in addition to providing comprehensive compliance dynamics in real-time, PVA-derived compliance offers a platform for investigating the pharmacological effects of reagents on bladder compliance, a parameter critically involved in urine storage.

## Data Availability

The original contributions presented in the study are included in the article/Supplementary Material, further inquiries can be directed to the corresponding author.

## Abbreviations

CON, control; IVP, intra-vesical pressure; IVV, intra-vesical volume; Cm, mean compliance; MB, mirabegron; OAB, overactive bladder; PBL, partial bladder ligation; PVA, pressure-volume analysis; SF, solifenacin; VEH, vehicle solution.
