# Supplementary figures and images for: Solifenacin/Mirabegron Induces an Acute Compliance Increase in the Filling Phase of the Capacity-Reduced Urinary Bladder: A Pressure-Volume Analysis in Rats
Source: Front Pharmacol. 2021 May 26;12:657959. doi: 10.3389/fphar.2021.657959 (PMC8188241; doi:10.3389/fphar.2021.657959)

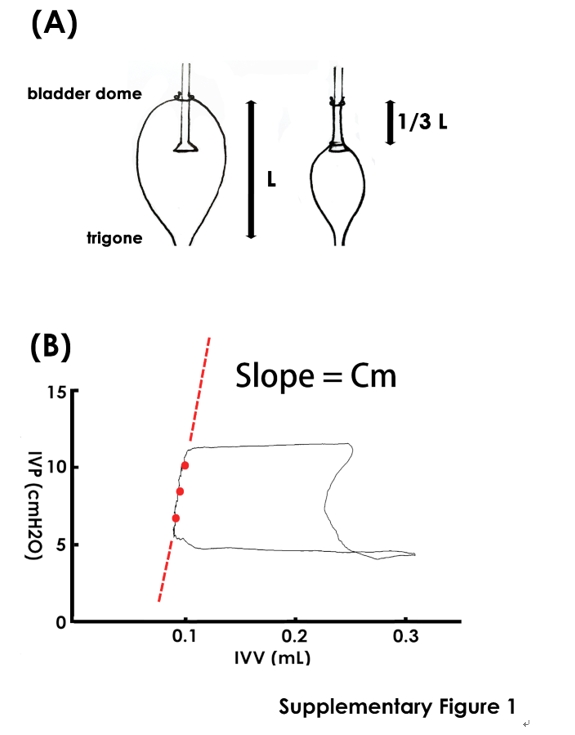

Supplement: Supplementary file 1 [file Image1.JPEG]
